# The Relationship Between Asian Dust Events and Out-of-Hospital Cardiac Arrests in Japan

**DOI:** 10.2188/jea.JE20140179

**Published:** 2015-04-05

**Authors:** Takahiro Nakamura, Masahiro Hashizume, Kayo Ueda, Tatsuhiko Kubo, Atsushi Shimizu, Tomonori Okamura, Yuji Nishiwaki

**Affiliations:** 1 Department of Environmental and Occupational Health, School of Medicine, Toho University, Tokyo, Japan; 1 東邦大学医学部社会学講座衛生学分野; 2 Department of Paediatric Infectious Diseases, Institute of Tropical Medicine, Nagasaki University, Nagasaki, Japan; 2 長崎大学熱帯医学研究所小児感染症学分野; 3 Department of Environmental Engineering, Kyoto University Graduate School of Engineering, Kyoto, Japan; 3 京都大学大学院工学研究科都市環境工学専攻 環境衛生学講座; 4 Department of Public Health, University of Occupational and Environmental Health, Fukuoka, Japan; 4 産業医科大学医学部公衆衛生学教室; 5 Regional Atmospheric Environment Section Center for Regional Environmental Research, National Institute for Environmental Studies, Tsukuba, Ibaraki, Japan; 5 独立行政法人国立環境研究所環境健康研究センター 広域大気環境研究室; 6 Department of Preventive Medicine & Public Heath, School of Medicine, Keio University, Tokyo, Japan; 6 慶應義塾大学医学部 衛生学公衆衛生学

**Keywords:** Asian dust, Utstein-Style data, out-of-hospital cardiac arrest, case crossover analysis

## Abstract

**Background:**

Asian dust events are caused by dust storms that originate in the deserts of China and Mongolia and drift across East Asia. We hypothesized that the dust events would increase incidence of out-of-hospital cardiac arrests by triggering acute events or exacerbating chronic diseases.

**Methods:**

We analyzed the Utstein-Style data collected in 2005 to 2008 from seven prefectures covering almost the entire length of Japan to investigate the effect of Asian dust events on out-of-hospital cardiac arrests. Asian dust events were defined by the measurement of light detection and ranging. A time-stratified case-crossover analysis was performed. The strength of the association between Asian dust events and out-of-hospital cardiac arrests was shown by odds ratios and 95% confidence intervals in two conditional logistic models. A pooled estimate was obtained from area-specific results by random-effect meta-analysis.

**Results:**

The total number of cases of out-of-hospital cardiac arrest was 59 273, of which 35 460 were in men and 23 813 were in women. The total number of event days during the study period was smallest in Miyagi and Niigata and largest in Shimane and Nagasaki. There was no significant relationship between Asian dust events and out-of-hospital cardiac arrests by area in either of the models. In the pooled analysis, the highest odds ratios were observed at lag day 1 in both model 1 (OR 1.07; 95% CI, 0.97–1.19) and model 2 (OR 1.08; 95% CI, 0.97–1.20). However, these results were not statistically significant.

**Conclusions:**

We found no evidence of an association between Asian dust events and out-of-hospital cardiac arrests.

## Abstract (ja)

**背景：:**

黄砂は中国とモンゴルの砂漠で巻き上げられた土壌粒子が偏西風により東アジアの広範囲に飛来し浮遊・降下する現象である。この黄砂が急性イベントや慢性疾患の増悪等を介して引き起こされる院外心停止との関連について検討を行った。

**方法：:**

黄砂の院外心停止への影響を調べるため、日本の南北に分布する7県から2005年から2007年までの4年間のウツタイン統計データを解析した。黄砂はLIDARデータによって定義した。解析方法にはTime-Stratifiedケースクロスオーバー解析を用いた。2つの条件付きロジスティックモデルにより、黄砂曝露と院外心停止との関連の強さはオッズ比とその95%信頼区間により示した。さらにメタアナリシス解析により7件を統合した結果を得た。

**結果：:**

全体の院外心停止件数は59,273件でそのうち35,460件が男性、23,813件が女性であった。対象期間の黄砂日は宮城と新潟で少なく、島根と長崎で多かった。黄砂と院外心停止は2つのモデルを用いて評価を行ったが有意な関連は得られなかった。統合した解析によるとModel1とModel2においてlag1で最も高いオッズ比を得た。しかしながらこれらの結果は統計学的に有意ではなかった。

**結論：:**

黄砂曝露と院外心停止において関連は認められなかった。

## INTRODUCTION

Asian dust events are caused by dust storms that originate in the deserts of China and Mongolia and drift across East Asia. They have been recognized as a worldwide environmental problem resulting from deforestation and desertification.^1^ The average particle diameter of Asian dust arriving in Japan is approximately 4 µm, but larger particles are also present, and it has been reported that sulfate and nitrate are sometimes attached to the surface of the dust particles.^2^ In addition, concerns have been raised that the microorganisms attached to the dust may cause allergic reactions^3^ and that the dust events may increase the incidence of respiratory and cardiovascular disease.^1^^,^^4^^,^^5^

Studies of the effects of Asian dust on mortality have been carried out in South Korea and Taiwan. Chen et al reported a 4.92% increase in total deaths in the 2 days following an Asian dust event in Taipei City.^6^ According to a report by Kwon et al, the estimated increase in all-cause mortality 2 days after Asian dust events in Korea was 3.4% overall and 5.3% among people aged 65 years or older.^7^^,^^8^ Some studies suggest that the effects of Asian dust may vary according to its components.^9^ To clarify these issues, evidence must be collected from various countries in Asia.

Only a few studies have investigated the association between Asian dust and mortality in Japan.^10^^,^^11^ Kashima et al performed a time-series analysis of mortality data collected between March 2005 and December 2010 and reported that a mean increase of 10 µg/m^3^ in Asian dust concentration at a lag of 2 days resulted in increases in mortality, including increases in mortality rates of 0.6% from heart disease, 0.8% from ischemic heart disease, 2.1% from arrhythmia, and 0.5% from pneumonia.^10^ As the study areas only covered prefectures in western Japan, wider-ranging studies are needed.

The Japanese Fire and Disaster Management Agency (FDMA) started collecting Utstein-Style data (a prospective, nation-wide, population-based registry system of out-of-hospital cardiac arrest in infants, children, and adults) in January 2005.^12^^,^^13^ We analyzed the data to investigate the effect of Asian dust events on cardiac arrests. We hypothesized that the events would increase incidence of out-of-hospital cardiac arrests by triggering acute events or exacerbating chronic diseases.

## MATERIALS AND METHODS

### Out of hospital cardiac arrest data

**Figure 1.**
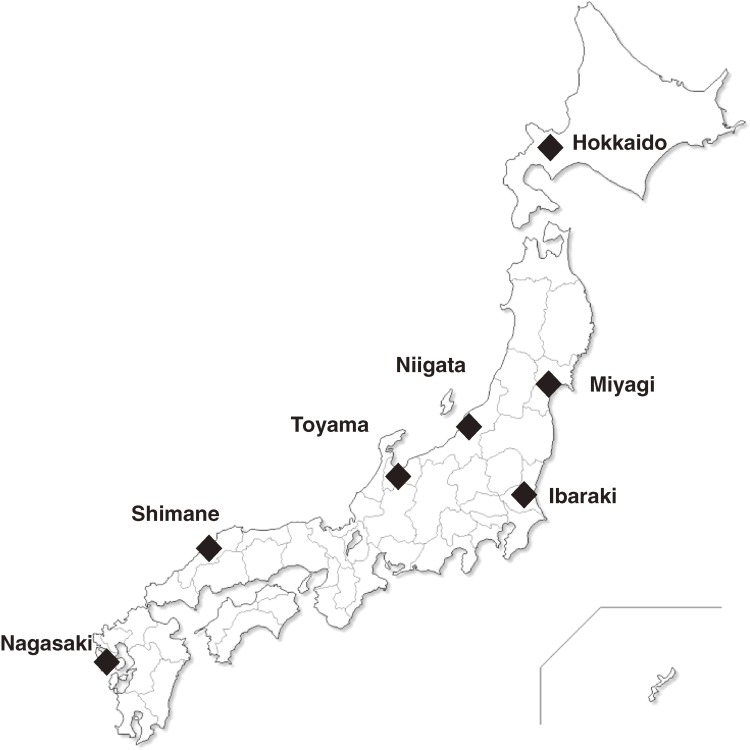
Study areas.

The FDMA’s All-Japan Utstein registry covers all 47 prefectures in Japan. For our study, we used data from seven prefectures covering almost the entire length of Japan, Hokkaido, Miyagi, Ibaraki, Niigata, Toyama, Shimane, and Nagasaki, where Asian dust is measured by light detection and ranging (LIDAR) and Asian dust data is prevalent during the study period (Figure 1). The Utstein-Style data include the onset date of arrest, the age and sex of the patient (but no identifying information), and the causes of arrest (cardiogenic or non-cardiogenic) determined by the doctor in charge.^13^ We used data for the 4 years between January 1, 2005, and December 31, 2008. The study was approved by the Ethics Committee of the School of Medicine, Toho University.

### Measurement of Asian dust

In general, air pollution particles secondarily generated from anthropogenic gases in the atmosphere, such as sulfate or nitrate, are spherical, but Asian dust particles are not. Because LIDAR can recognize shape differences by utilizing information about the polarization of scattered light, it is possible to measure non-spherical Asian dust particles separately from the spherical particles of other air pollutants. Therefore, LIDAR has been widely used in observations of Asian dust events in East Asian countries. The National Institute for Environmental Studies LIDAR network can estimate the dust extinction coefficients of the non-spherical and spherical components.^14^

The dust extinction coefficient is estimated in 30-meter increments between 120 m and 6000 m above the ground every 15 minutes. If the lower atmosphere is well mixed, the concentration of Asian dust on the ground is similar to that between 120 m and 270 m, the lowest level for which LIDAR data are available. Utstein-Style data are the sum of 24-hour measurements (from midnight to midnight), and LIDAR measurements of Asian dust are also compiled as total 24-hour data (midnight to midnight). Asian dust extinction coefficients larger than 1/km and spherical extinction coefficients larger than 2/km were excluded because the data may have been influenced by meteorological factors, such as cloud, fog, rain, and snow.

In our study, data from LIDAR measurements of Asian dust had to satisfy the following criteria to be considered valid^15^:

1. LIDAR data were available at least 20 hours per day.
2. The correlation coefficient between the hourly Asian dust extinction coefficient and hourly suspended particulate matter (SPM) concentration was more than 0.6.
3. The daily maximum SPM concentration was over 50 µg/m^3^.
4. The daily maximum Asian dust extinction as measured by LIDAR was more than 0.05/km.

LIDAR was set up at one point in each prefecture. There are gaps in the LIDAR data between January and October 2005 for Miyagi, between January 2005 and March 2007 for Niigata, between January and March 2005 for Shimane, and before March 2006 for Nagasaki.

For SPM concentrations, we used the daily average of hourly measurements made by air pollution monitoring stations in each study area. SPM is defined under the Japanese Air Quality Standard as any particle collected with an upper 100% cut-off point of 10 µm in aerodynamic diameter.^16^ The 50% cut-off diameter for SPM is assumed to be approximately 7 µm.

### Other air pollutants and meteorological data

Data on other air pollutants (SO_2_, NO_2_, and Ox [O_3_ + NO_2_]) were collected from air pollution monitoring stations in each study area (99 stations in Hokkaido, 38 in Miyagi, 52 in Ibaraki, 32 in Niigata, 30 in Toyama, 9 in Shimane, and 21 in Nagasaki). Daily average concentrations of SO_2_ and NO_2_ were calculated from hourly concentrations measured at each station and averaged. In the case of Ox, maximum hourly concentrations measured at each station were used. Data based on less than 20 hourly measurements on any 1 day were excluded.

Daily temperature and relative humidity data were obtained from the Japanese Meteorological Agency. For each area, we calculated the daily average of temperature and relative humidity from data collected hourly (between 00:00 and 23:00) and recorded the maximum and minimum (temperatures) in each area.

### Statistical analysis

A time-stratified case-crossover analysis was performed to assess the relationship between Asian dust events and out-of-hospital cardiac arrests. In the same way as a matched case-control analysis, this analysis assigned the day on which an out-of-hospital cardiac arrest occurred as the case day and comparisons were made with control days chosen on the same day of the week earlier and later in the same month in the same year.^17^ The advantage of this design is that it controls for the effects of long-term trends, seasonality, and the day of the week. The strength of the association between Asian dust events and out-of-hospital cardiac arrests was shown using odds ratios (ORs) and 95% confidence intervals (CIs) of two conditional logistic models. Model 1 included temperature and relative humidity (averages of the case day, lag day 1, and lag day 2, smooth spline [degree of freedom = 6]) as co-variables, with Asian dust events as the main exposure variable. Model 2 included the same variables, along with SO_2_, NO_2_, and Ox concentrations (averages on the case day, lag day 1, and lag day 2) and the spherical extinction coefficients (average on the case day, lag day 1, and lag day 2).

Because the effects of Asian dust events can persist over several days, we examined the effects with several lag times (lag day 0, lag day 1, lag day 2, and lag day 3) and also the cumulative effects of lag days 0–3. Three case definitions were used after excluding cardiac arrest caused by external factors, including trauma:

1. Cardiac arrest from all causes
2. Cardiac arrest from cardiogenic causes
3. Cardiac arrest from non-cardiac causes

A pooled estimate was obtained from area-specific results via random-effect meta-analysis. This analysis was repeated after stratification by sex. We also repeated the analysis by season. As most Asian dust events occurred in spring, however, we could not construct valid statistical models for seasons other than spring, so we examined the association between Asian dust and cardiac arrests in spring (March through May). All analyses were performed with STATA ver. 12 (Stata Corp, College Station, TX, USA).

## RESULTS

The total number of cases of out-of-hospital cardiac arrest was 59 273, of which 35 460 were in men and 23 813 were in women. Table 1 shows the cases in the seven areas, classified according to sex, age category, and year. The largest number of cases (18 539) was reported in Hokkaido, and the smallest number cases (3339) was reported in Shimane. In all prefectures, patients were likely to be male and over 50 years of age. There was no significant difference in the number of cases by year.

**Table 1.** Demographic characteristics of sudden out-of-hospital cardiac arrests

	Hokkaido	Miyagi	Ibaraki	Niigata	Toyama	Shimane	Nagasaki
Total number	18 539	8648	10 413	10 017	3942	3339	4375
Sex							
Male	11 182	5041	6316	5916	2386	1928	2691
Female	7357	3607	4097	4101	1556	1411	1684
Age							
0–9	286	102	127	82	35	43	59
10–19	169	74	108	74	25	32	39
20–29	460	175	212	133	61	43	80
30–39	594	240	317	223	84	79	130
40–49	978	386	438	352	141	115	200
50–59	2046	771	1005	805	364	301	475
60–69	3035	1151	1553	1207	567	420	614
70–79	4623	2177	2534	2628	988	838	1148
80–	6341	3572	4117	4498	1676	1468	1630
Year							
2005	4524	2103	2573	2443	844	742	1010
2006	4532	2062	2458	2407	1019	821	1054
2007	4647	2207	2585	2610	967	887	1149
2008	4836	2276	2797	2557	1112	889	1162

Table 2 shows the distribution of dust extinction coefficients; spherical extinction coefficients; SPM, NO_2_, SO_2_, and Ox levels; and meteorological data, including average temperature, maximum temperature, minimum temperature, and humidity, by area. During the study period, both the average and median of the dust extinction coefficient were highest in Shimane (0.032/km and 0.024/km, respectively) and lowest in Miyagi (0.011/km and 0.006/km, respectively). The average SPM level was highest in Nagasaki (28.7 µg/m^3^) and lowest in Hokkaido (14.4 µg/m^3^).

**Table 2.** Levels of dust and air pollutants and meteorological observations during the study period

	Days	Mean	SD	25th percentile	Median	75th percentile
Dust extinction coefficients (/km)						
Hokkaido	1199	0.012	0.014	0.003	0.008	0.015
Miyagi	873	0.011	0.016	0.003	0.006	0.012
Ibaraki	1333	0.017	0.014	0.007	0.016	0.025
Niigata	529	0.021	0.026	0.008	0.015	0.025
Toyama	1122	0.015	0.022	0.005	0.010	0.017
Shimane	1159	0.032	0.035	0.016	0.024	0.036
Nagasaki	1103	0.018	0.024	0.005	0.011	0.022
Spherical extinction coefficients (/km)						
Hokkaido	1199	0.087	0.077	0.036	0.068	0.115
Miyagi	870	0.074	0.107	0.014	0.039	0.085
Ibaraki	1333	0.120	0.097	0.050	0.095	0.162
Niigata	529	0.133	0.095	0.051	0.122	0.199
Toyama	1122	0.059	0.061	0.016	0.045	0.083
Shimane	1159	0.112	0.077	0.056	0.097	0.152
Nagasaki	1103	0.086	0.071	0.038	0.069	0.115
SPM (µg/m^3^)						
Hokkaido	1461	14.4	6.6	10.1	12.6	16.6
Miyagi	1461	21.2	11.4	13.2	18.4	26.4
Ibaraki	1461	23.8	12.7	14.7	20.7	29.7
Niigata	1461	22.2	11.5	13.9	19.8	27.5
Toyama	1461	19.9	11.6	11.4	16.9	25.4
Shimane	1461	23.5	13.4	14.3	20.5	29.2
Nagasaki	1461	28.7	14.7	18.6	24.9	34.7
NO_2_ (ppb)						
Hokkaido	1461	11.4	4.8	7.9	10.3	14.0
Miyagi	1461	13.3	4.8	9.9	12.5	16.2
Ibaraki	1461	12.2	4.7	8.8	11.2	14.9
Niigata	1461	10.5	4.3	7.5	9.9	12.6
Toyama	1461	11.5	4.3	8.5	10.9	14.0
Shimane	1461	6.4	2.5	4.6	6.0	7.7
Nagasaki	1461	11.3	3.9	8.5	10.9	14.0
SO_2_ (ppb)						
Hokkaido	1461	3.1	0.7	2.6	3.1	3.5
Miyagi	1461	1.0	0.5	0.7	0.9	1.2
Ibaraki	1461	3.4	1.0	2.7	3.3	3.9
Niigata	1461	2.0	0.8	1.5	1.9	2.4
Toyama	1461	1.8	0.7	1.4	1.7	2.2
Shimane	1461	1.4	1.2	0.7	1.1	1.7
Nagasaki	1461	3.3	1.1	2.5	3.1	3.9
Ox (ppb)						
Hokkaido	1461	38.2	9.5	31.9	36.7	43.8
Miyagi	1461	44.1	12.0	36.5	42.4	50.9
Ibaraki	1461	49.4	16.0	38.7	46.1	57.0
Niigata	1461	48.5	12.0	40.4	46.4	55.2
Toyama	1461	51.7	13.5	41.8	49.6	60.4
Shimane	1461	50.8	13.5	41.3	48.3	59.2
Nagasaki	1461	47.5	15.1	38.2	46.2	57.6
Temperature Average (°C)						
Hokkaido	1461	9.3	9.5	0.5	9.6	18.0
Miyagi	1461	12.6	8.2	5.0	12.9	19.5
Ibaraki	1461	14.0	8.0	6.6	14.4	20.9
Niigata	1461	14.1	8.6	6.2	14.1	21.8
Toyama	1461	14.5	8.8	6.7	14.9	22.0
Shimane	1461	15.2	8.3	7.8	15.4	22.2
Nagasaki	1461	17.6	7.8	11.0	18.0	24.6
Temperature Max (°C)						
Hokkaido	1461	13.2	10.4	3.3	14.0	22.5
Miyagi	1461	16.6	8.4	9.3	17.4	23.3
Ibaraki	1461	18.9	7.7	12.2	19.2	25.1
Niigata	1461	17.8	9.0	9.8	18.4	25.5
Toyama	1461	19.0	9.3	10.9	19.9	26.5
Shimane	1461	19.8	8.7	12.3	20.6	26.8
Nagasaki	1461	21.4	7.9	14.7	22.1	28.1
Temperature Min (°C)						
Hokkaido	1461	5.8	9.4	−2.6	5.4	14.2
Miyagi	1461	9.2	8.5	1.0	8.8	16.9
Ibaraki	1461	9.7	8.9	1.4	9.8	17.7
Niigata	1461	10.9	8.6	2.9	10.6	19.0
Toyama	1461	10.7	8.6	2.6	10.3	18.8
Shimane	1461	11.5	8.5	4.1	10.9	19.1
Nagasaki	1461	14.2	8.1	6.8	14.1	21.8
Humidity (%)						
Hokkaido	1461	68.0	10.0	62	68	75
Miyagi	1461	72.6	13.2	63	73	83
Ibaraki	1461	72.6	12.1	65	75	82
Niigata	1461	69.5	8.9	64	70	76
Toyama	1461	78.6	10.2	73	79	86
Shimane	1461	73.9	8.9	69	74	80
Nagasaki	1461	67.2	11.6	59	67	76

SD, standard deviation; SPM, suspended particulate matter.

Table 3 shows the number of Asian dust events defined by LIDAR by areas and year. The total number of event days during the study period was smallest in Miyagi and Niigata and largest in Shimane and Nagasaki.

**Table 3.** Number of Asian dust events by LIDAR measurement

	2005	2006	2007	2008	Total
Hokkaido	0	6	3	2	11
Miyagi	2	1	1	3	7
Ibaraki	2	9	12	1	24
Niigata	—	—	4	4	8
Toyama	4	7	7	3	21
Shimane	19	24	24	12	79
Nagasaki	—	19	18	10	47

Table 4 shows the association between Asian dust events and out-of-hospital cardiac arrests (combined cardiogenic and non-cardiogenic). There was no significant relationship between Asian dust events and out-of-hospital cardiac arrests in either model 1 or model 2. In the pooled analysis, the highest ORs were observed at lag day 1 in both model 1 (OR 1.07; 95% CI, 0.97–1.19) and model 2 (OR 1.08; 95% CI, 0.97–1.20). However, these results were not statistically significant. We repeated the analysis and examined the association between Asian dust and cardiac arrests in spring and found no association (eTable).

**Table 4.** Odds ratios and 95% confidence intervals for total sudden cardiac arrests (excluding external causes) caused by dust exposure (binary), as assessed by LIDAR measurements (men and women)

	Climatically adjusted odds ratios (95% CI) (model 1)					Odds ratios (95% CI), further adjusted by air pollution (model 2)				
	
	Lag day 0	Lag day 1	Lag day 2	Lag day 3	Lag days 0–3	Lag day 0	Lag day 1	Lag day 2	Lag day 3	Lag days 0–3
Total excluding external cause										
Hokkaido	0.957 (0.760–1.206)	1.071 (0.838–1.370)	0.958 (0.741–1.238)	0.957 (0.744–1.233)	1.048 (0.895–1.226)	0.957 (0.759–1.207)	1.070 (0.835–1.369)	0.950 (0.735–1.228)	0.951 (0.738–1.224)	1.050 (0.886–1.244)
Miyagi	1.181 (0.725–1.927)	1.501 (0.881–2.556)	1.238 (0.732–2.091)	1.129 (0.728–1.750)	1.211 (0.7339–1.998)	1.085 (0.642–1.832)	1.371 (0.761–2.468)	1.009 (0.56–1.821)	1.152 (0.743–1.786)	1.675 (0.830–3.380)
Ibaraki	1.017 (0.829–1.247)	1.035 (0.833–1.285)	1.037 (0.837–1.285)	1.069 (0.872–1.311)	1.056 (0.907–1.228)	1.030 (0.839–1.265)	1.063 (0.854–1.322)	1.022 (0.823–1.270)	1.058 (0.862–1.298)	1.080 (0.926–1.258)
Niigata	0.636 (0.385–1.050)	0.904 (0.534–1.53)	0.730 (0.429–1.242)	0.721 (0.417–1.247)	0.732 (0.533–1.004)	0.632 (0.378–1.058)	0.981 (0.556–1.729)	0.832 (0.479–1.444)	0.760 (0.435–1.326)	0.768 (0.541–1.090)
Toyama	1.007 (0.680–1.492)	1.124 (0.770–1.641)	0.835 (0.565–1.235)	1.107 (0.761–1.611)	0.947 (0.712–1.260)	1.019 (0.678–1.530)	1.016 (0.675–1.531)	0.817 (0.549–1.216)	1.121 (0.770–1.631)	0.872 (0.626–1.215)
Shimane	0.920 (0.735–1.152)	1.124 (0.908–1.391)	0.980 (0.789–1.218)	0.922 (0.744–1.142)	1.009 (0.872–1.167)	0.995 (0.787–1.258)	1.199 (0.957–1.502)	1.008 (0.807–1.260)	0.904 (0.728–1.123)	1.055 (0.908–1.226)
Nagasaki	1.008 (0.805–1.262)	1.014 (0.803–1.279)	0.993 (0.785–1.257)	0.838 (0.659–1.065)	0.928 (0.818–1.053)	0.985 (0.783–1.238)	0.979 (0.771–1.243)	0.980 (0.769–1.250)	0.838 (0.658–1.066)	0.891 (0.778–1.021)

Pooled analysis	0.970 (0.876–1.073)	1.074 (0.968–1.191)	0.979 (0.881–1.088)	0.962 (0.868–1.066)	0.986 (0.918–1.059)	0.981 (0.884–1.088)	1.078 (0.968–1.201)	0.973 (0.874–1.084)	0.958 (0.864–1.063)	0.992 (0.900–1.093)

CI, confidence interval.

**Figure 2.**
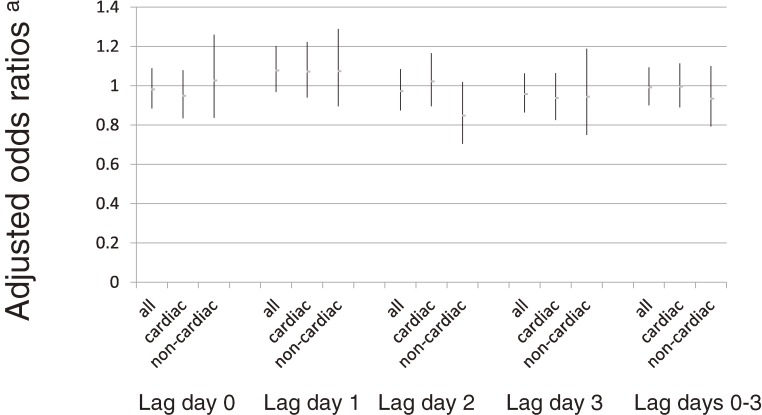
Odds ratios further adjusted for air pollution (men and women combined). ^a^Vertical bar indicates 95% confidence intervals of odds ratios.

**Figure 3.**
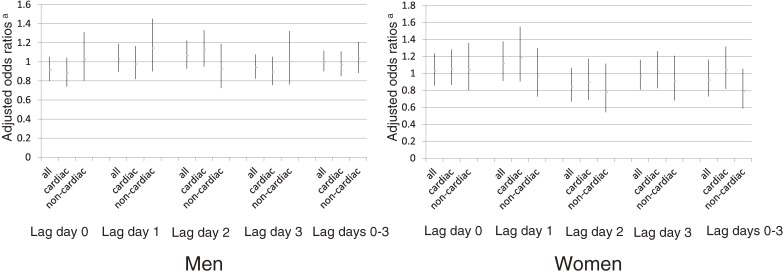
Odds ratios further adjusted for air pollution (Left: men, Right: women). ^a^Vertical bar indicates 95% confidence intervals of odds ratios.

Figure 2 and Figure 3 show the results of a pooled analysis of all out-of-hospital cardiac arrests, cardiogenic cardiac arrests, and non-cardiogenic cardiac arrests using model 2 ORs; Figure 2 shows the results for men and women combined, and Figure 3 shows the results according to sex. None of the results show any significant relationship between Asian dust events and outcomes. When we limited our analysis to subjects aged over 65 years, we obtained similar results (data not shown).

## DISCUSSION

In this study, we found no statistically significant relationship between Asian dust events and out-of-hospital cardiac arrests. In contrast, a study carried out in Taipei by Chen and Yang estimated a dust storm-related increase of 0.48% in the number of total deaths on the day of the storm itself and increases of 0.41% and 4.92%, respectively, on the following 1 and 2 days.^6^ However, the observed effects did not reach statistical significance. In Korea, Hwang reported estimated increases in all-cause mortality of 2.5% overall and 2.2% among those aged 65 years and older.^7^ Also in Korea, Kwon reported an estimated increase in total deaths of 3.4% on the 2 days following a storm and an increase of 5.3% among those ages for 65 years and older.^8^ In Japan, Ueda reported a 12.1% increase in ambulance dispatches due to all causes (95% CI, 2.3%–22.9%), and a 20.8% increase due to cardiovascular diseases (95% CI, 3.5%–40.9%) at lag days 0–3 from days when heavy Asian dust events occurred.^18^

Although the mechanisms of any link between Asian dust and cardiac arrests remain unclear, possible biologic mechanisms include the deterioration of underlying health conditions involving circulatory diseases, such as arrhythmia, and respiratory diseases, such as bronchial asthma. Asian dust particles contain rock-forming minerals, such as quartz and feldspar, and argillites, such as mica, kaolinite, and green-mud stones. However, sulfur oxidants, nitrogen oxidants, ammonium, and microorganisms are also attached to Asian dust particles, and these may have effects on health,^19^^,^^20^ as inhaling them can cause allergic respiratory reactions.^3^

We hypothesized that the effects of Asian dust would be stronger outside air-conditioned environments like hospitals, but our results did not show this. One possible reason for the discrepancy between our results and those of previous studies showing positive associations between Asian dust events and health effects is differences in the definitions of Asian dust used for the exposure index. While Asian dust was defined on the basis of PM_10_ concentrations in the Taiwanese and Korean studies,^6^^–^^8^ Japanese studies use the meteorological office’s naked-eye observation-based declaration of dust events or LIDAR measurements, as we did. The definition based on PM_10_ concentrations includes daily average PM_10_ concentrations above specified cut-off levels,^6^ measurements of concentrations exceeding 100 µg/m^3^, and average concentration increases exceeding 50 µg/m^3^.^21^ However, the definition of dust events based on PM_10_ concentrations can be problematic, as PM_10_ contains particles from other forms of air pollution, so measurements do not necessarily indicate Asian dust-particle concentration.

Our study relied on LIDAR data, in which Asian dust particles and other air pollutants are measured independently using depolarization ratios, making LIDAR a more reliable method for directly assessing Asian dust levels. However, we were unable to obtain LIDAR measurements any lower than 120 meters above the ground, so we cannot discount the possibility that the concentrations of the particles people actually inhaled were different. Also, because the Utstein data were summarized by area, we applied a single exposure level for each area. This potential exposure misclassification could have weakened the statistical power of our study, preventing us from detecting an association between Asian dust and cardiac arrests. In addition, misclassification of other air pollutant concentrations is possible. Although the concentrations would be better based on the levels at the monitoring stations nearest to the subjects’ residence, it was impossible to gather such data because we could not obtain their address information due to privacy protection.

A second possible explanation for the discrepancy between our results and those of previous studies may be the different outcome criteria. We used out-of-hospital cardiac arrest as our outcome variable. Out-of-hospital cardiac arrest was defined as the cessation of cardiac mechanical activity outside the hospital, as confirmed by the absence of signs of circulation. Even if exposure to Asian dust caused circulatory problems in individual subjects, these would not be included in the Ustein data unless cardiac arrest occurred before the subject arrived at the hospital, as cases of cardiac arrest occurring in the hospital were not included in the Utstein data.

The Ustein data cover all prefectures in Japan and offer almost complete enumeration of relevant cases. The data are available to all researchers working in the field of environmental epidemiology, and because the data format of the Ustein registry is internationally standardized, the registry can be used worldwide for epidemiological studies.

### Conclusions

We found no evidence of an association between Asian dust events and out-of-hospital cardiac arrests.

## ONLINE ONLY MATERIALS

eTable. Odds ratios and 95% confidence intervals of total sudden cardiac arrests (excluding external cause) for dust exposure (binary), as assessed by LIDAR measurements during spring (men and women).

Abstract in Japanese.
