# Supplementary material for: The Relationship Between Asian Dust Events and Out-of-Hospital Cardiac Arrests in Japan
Source: J Epidemiol. 2015 Apr 5;25(4):289–96. doi: 10.2188/jea.JE20140179 (PMC4375283; doi:10.2188/jea.JE20140179)
Supplement: eTable. [file je-25-289-s001.pdf]

eTable. Odds ratios and 95% confidence intervals of total sudden cardiac arrests (excluding external cause) for dust exposure (binary), as assessed by LIDAR measurements during spring season (men and women)

	Climatically adjusted odds ratios (95% CI) (model 1)					Odds ratios (95% CI), further adjusted by air pollution (model 2)				
	Lag day 0	Lag day 1	Lag day 2	Lag day 3	Lag days 0-3	Lag day 0	Lag day 1	Lag day 2	Lag day 3	Lag days 0-3
**Total excluding external cause**										
Hokkaido	0.957 (0.723-1.265)	1.081 (0.821-1.423)	0.882 (0.649-1.199)	1.080 (0.814-1.434)	1.059 (0.891-1.259)	0.972 (0.730-1.293)	1.085 (0.818-1.438)	0.895 (0.657-1.219)	1.065 (0.801-1.416)	1.027 (0.845-1.249)
Miyagi	1.164 (0.659-2.058)	1.658 (0.868-3.166)	1.034 (0.562-1.903)	1.248 (0.731-2.131)	1.569 (0.744-3.311)	0.913 (0.460-1.811)	1.542 (0.694-3.427)	0.670 (0.318-1.412)	1.292 (0.742-2.250)	2.579 (0.546-12.189)
Ibaraki	0.858 (0.602-1.221)	1.125 (0.777-1.629)	1.178 (0.818-1.695)	0.990 (0.703-1.394)	1.055 (0.809-1.376)	0.890 (0.619-1.280)	1.126 (0.767-1.652)	1.122 (0.763-1.651)	1.019 (0.714-1.454)	0.955 (0.709-1.285)
Niigata	0.748 (0.366-1.528)	0.710 (0.322-1.566)	0.577 (0.268-1.240)	0.743 (0.386-1.430)	0.734 (0.527-1.021)	0.738 (0.352-1.545)	0.757 (0.319-1.797)	0.597 (0.270-1.320)	0.735 (0.372-1.453)	0.710 (0.484-1.040)
Toyama	0.615 (0.396-0.954)	1.023 (0.700-1.496)	0.925 (0.639-1.340)	0.988 (0.675-1.446)	0.792 (0.574-1.094)	0.673 (0.423-1.072)	1.328 (0.857-2.060)	1.065 (0.717-1.581)	0.986 (0.672-1.446)	0.634 (0.401-1.002)
Shimane	1.004 (0.819-1.23)	0.899 (0.726-1.114)	1.068 (0.869-1.312)	1.176 (0.962-1.437)	1.069 (0.928-1.231)	1.113 (0.894-1.384)	0.937 (0.740-1.186)	1.104 (0.891-1.369)	1.168 (0.952-1.432)	1.123 (0.965-1.307)
Nagasaki	0.930 (0.72-1.201)	0.847 (0.644-1.114)	0.847 (0.648-1.106)	1.073 (0.832-1.384)	0.908 (0.795-1.036)	0.918 (0.701-1.202)	0.798 (0.594-1.071)	0.844 (0.635-1.123)	1.109 (0.856-1.436)	0.908 (0.778-1.059)
Pooled analysis	0.926 (0.822-1.043)	0.973 (0.861-1.100)	0.967 (0.856-1.092)	1.084 (0.965-1.218)	0.972 (0.872-1.084)	0.957 (0.844-1.086)	1.000 (0.870-1.149)	0.976 (0.859-1.110)	1.092 (0.970-1.229)	0.946 (0.822-1.089)

CI, confidence interval.
