# Supplementary material for: The Relationship Between Asian Dust Events and Out-of-Hospital Cardiac Arrests in Japan
Source: J Epidemiol. 2015 Apr 5;25(4):289–96. doi: 10.2188/jea.JE20140179 (PMC4375283; doi:10.2188/jea.JE20140179)
Supplement: Abstract in Japanese. [file je-25-289-s002.pdf]

ウツタイン統計を用いた黄砂と院外心停止との関連

中村　孝裕[1)]、橋爪　真弘[2)]、上田　佳代[3)]、久保　達彦[4)]、清水　厚[5)]、岡村　智教[6)]、西脇　祐司[1)]

1) 東邦大学医学部社会学講座衛生学分野

2) 長崎大学熱帯医学研究所小児感染症学分野

3) 京都大学大学院工学研究科都市環境工学専攻　環境衛生学講座

4) 産業医科大学医学部公衆衛生学教室

5) 独立行政法人国立環境研究所環境健康研究センター　広域大気環境研究室

6) 慶應義塾大学医学部　衛生学公衆衛生学

背景：黄砂は中国とモンゴルの砂漠で巻き上げられた土壌粒子が偏西風により東アジアの広範囲に飛来し浮遊・降下する現象である。この黄砂が急性イベントや慢性疾患の増悪等を介して引き起こされる院外心停止との関連について検討を行った。

方法：黄砂の院外心停止への影響を調べるため、日本の南北に分布する 7 県から 2005 年から 2007 年までの 4 年間のウツタイン統計データを解析した。黄砂は LIDAR データによって定義した。解析方法には Time-Stratified ケースクロスオーバー解析を用いた。2 つの条件付きロジスティックモデルにより、黄砂曝露と院外心停止との関連の強さはオッズ比とその 95%信頼区間により示した。さらにメタアナリシス解析により 7 件を統合した結果を得た。

結果：全体の院外心停止件数は 59,273 件でそのうち 35,460 件が男性、23,813 件が女性であった。対象期間の黄砂日は宮城と新潟で少なく、島根と長崎で多かった。黄砂と院外心停止は 2 つのモデルを用いて評価を行ったが有意な関連は得られなかった。統合した解析によると Model1 と Model2 において lag1 で最も高いオッズ比を得た。しかしながらこれらの結果は統計学的に有意ではなかった。

結論：黄砂曝露と院外心停止において関連は認められなかった。

キーワード：黄砂、ウツタイン統計データ、院外心停止、ケースクロスオーバー解析
